# Trials and tribulations of S49 orders

**DOI:** 10.1192/bjb.2018.108

**Published:** 2019-02

**Authors:** Ilyas Mirza, Mukesh Kripalani

**Affiliations:** Consultant Psychiatrist, Barnet Enfield and Haringey Mental Health NHS Trust; email: ilyasmirza@nhs.net

The Mental Capacity Act 2005 (MCA 2005) is an Act of Parliament, applying to England and Wales, that provides a legal framework for acting and making decisions on behalf of adults who lack the capacity to make particular decisions for themselves.^1^ Under section 49 (pilot order) of the MCA 2005, launched in 2016, the Court of Protection can order reports from National Health Service (NHS) health bodies and local authorities when it is considering any question relating to someone who may lack capacity, and the report must deal with ‘such matters as the court may direct’.^2^ This change has caused significant ethical challenges for psychiatrists.

With regard to professional implications, Section 49 reports require an opinion; according to British Medical Association (BMA) and General Medical Council (GMC) guidance, this falls under expert witness work. The recent Pool judgment is a reminder that the GMC is likely to consider that fitness to practice is impaired if a doctor acts outside what is considered their scope of work.^3^ The order is usually accompanied by an instruction letter containing legal precedents and a bundle sometimes containing conflicting assessments. Responding to such instructions require medico-legal training and experience in giving opinions to complex questions such as capacity to consent to sex, or consent to drink. We would argue that there is a blurring of boundaries between expert and professional witness. There is a need to clarify what legal safeguards are in place for the author of Section 49 reports, if their opinion is challenged, as it was in the Pool case.

In relation to patient care, the introduction of an automatic right to a medico-legal report, which was previously funded from elsewhere, has shifted the cost on to the NHS. Given that mental health services are still block funded; more work without additional funding leads to dilution of quality of care elsewhere in the system, affecting patient care. Lack of parity of esteem between physical and mental health funding makes this work an onerous burden. Increased workload without remuneration has an adverse effect on staff morale, influencing recruitment and retention within an already struggling NHS.

There is an urgent need to quantify the effects of these orders on services. The Royal College of Psychiatrists, working together with NHS England and the BMA, needs to define how medico-legal work can be safely done within existing resources. Moreover, the BMA, GMC, the College and NHS employers need to resolve the discrepancy that results from what is considered expert witness work by regulatory bodies being framed as normal NHS work by the Court of Protection.^4^ Legal safeguards need to be in place if NHS professionals become subject to legal challenge, e.g. from an aggrieved solicitor. Consideration needs to be given to a fresh legal challenge if it is evident that this pilot order is affecting patient care.
